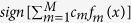# Non-invasive optical estimate of tissue composition to differentiate malignant from benign breast lesions: A pilot study

**DOI:** 10.1038/srep40683

**Published:** 2017-01-16

**Authors:** Paola Taroni, Anna Maria Paganoni, Francesca Ieva, Antonio Pifferi, Giovanna Quarto, Francesca Abbate, Enrico Cassano, Rinaldo Cubeddu

**Affiliations:** 1 Dipartimento di Fisica, Politecnico di Milano, Piazza Leonardo da Vinci 32, 20133 Milano, Italy; 2 Istituto di Fotonica e Nanotecnologie, Consiglio Nazionale delle Ricerche, Piazza Leonardo da Vinci 32, 20133 Milano, Italy; 3 Dipartimento di Matematica, Politecnico di Milano, Piazza Leonardo da Vinci 32, 20133 Milano, Italy; 4 Dipartimento di Matematica, Università degli Studi, Via Saldini 50, 20133 Milano, Italy; 5 European Institute of Oncology, Breast Imaging Unit, Via G. Ripamonti, 435, 20141 Milano, Italy

## Abstract

Several techniques are being investigated as a complement to screening mammography, to reduce its false-positive rate, but results are still insufficient to draw conclusions. This initial study explores time domain diffuse optical imaging as an adjunct method to classify non-invasively malignant *vs* benign breast lesions. We estimated differences in tissue composition (oxy- and deoxyhemoglobin, lipid, water, collagen) and absorption properties between lesion and average healthy tissue in the same breast applying a perturbative approach to optical images collected at 7 red-near infrared wavelengths (635–1060 nm) from subjects bearing breast lesions. The Discrete AdaBoost procedure, a machine-learning algorithm, was then exploited to classify lesions based on optically derived information (either tissue composition or absorption) and risk factors obtained from patient’s anamnesis (age, body mass index, familiarity, parity, use of oral contraceptives, and use of Tamoxifen). Collagen content, in particular, turned out to be the most important parameter for discrimination. Based on the initial results of this study the proposed method deserves further investigation.

The International Agency for Research on Cancer (IARC) has recently confirmed the clear effectiveness of mammographic screening in reducing breast-cancer mortality (23% reduction in women 50 to 69 years of age)^1^,^2^. However, (film and digital) mammography has known limitations.

The main harms associated with early detection of breast cancer through mammographic screening are false positive results, overdiagnosis, and, possibly, radiation-induced cancer^1^. In particular, the cumulative risk of a false-positive mammogram over a 10-year period of yearly screening is on average of about 50 to 60 percent^3^,^4^,^5^,^6^,^7^. False-positive results were shown to cause anxiety, distress, and other psychosocial effects^8^. Still more critical, they can lead to additional imaging and even invasive procedures, like fine-needle aspiration and biopsy.

The chance of a false positive result is higher among young women and women with dense breasts (categories that often overlap)^4^. Over the years, the widespread use of screening has contributed to a significant reduction in breast cancer mortality. This notwithstanding, the higher false-positive rate at younger age impacted on the U.S. Preventive Services Task Force decision to recommend against routine screening of women 40 to 49 years of age^9^. This notwithstanding the fact the risk to develop breast cancer is lower at younger age, but cancer is more aggressive and responds less to therapy, leading to a high number of deaths (more than 15% of the overall number of deaths for breast cancer^9^).

Several techniques have been or are being investigated as an adjunct to mammography: tomosynthesis, ultrasonography (US), magnetic resonance imaging (MRI) with or without the administration of a contrast agent, and positron emission tomography (PET). Results from population screening on the use of other techniques as an addition to mammography are still insufficient to draw conclusions. However, it seems difficult to improve concurrently sensitivity and specificity. For example, adjunct US is useful to image dense breasts and it increases the rate of cancer detection. However, the evidence for reduction in breast-cancer mortality is still inadequate, while it yields a higher number of false positive detections^1^,^2^.

Optical techniques are inherently non-invasive, provide absolute operator-independent outcomes, and are comparatively cheap and easy to operate and maintain as compared to methods that are routinely applied for medical diagnosis. Over the last couple of decades, they have been explored as potential diagnostic means. In particular, diffuse optics allows the estimate of the absorption and reduced scattering properties of highly diffusive media, such as biological tissues. In turn, the knowledge of the absorption and reduced scattering provides information on tissue composition and microscopic structure, respectively. Most efforts have been devoted to the detection of breast cancer and to functional neuroimaging^10^,^11^,^12^. To this purpose, motivated by more readily available and cheaper instrumentation and supported by pathophysiological grounds, the spectral range of 650 to 850 nm was most often considered, focusing on blood parameters, like total hemoglobin content and oxygen saturation level, which are effectively estimated in that wavelength interval.

In general, higher blood content proved to be a good biomarker for cancer, while the situation is much more controversial with regard to the oxygenation level^13^,^14^. Higher hemoglobin content is in agreement with typical conditions in breast tumors, such as neovascularization, altered vascular morphology (*e.g.*, vascular tortuosity), and blood cell extravasation. Similarly, well-known breast tumor features may explain conflicting reports on the oxygen saturation level *SO*_*2*_ = *HbO*_*2*_/(*Hb* + *HbO*_*2*_). In particular, *SO*_*2*_ as measured by diffuse optical techniques is an estimate of the fraction of hemoglobin that binds oxygen within the macroscopic volume of tissue probed by the measurement. For compressed breast transmittance imaging, as performed in the present study, such volume corresponds to a fuse-like region, connecting the light injection and collection points on opposite breast surfaces and roughly measuring a few cubic centimeters. Thus, the estimated *SO*_*2*_ value provides information on macroscopic, but still local environment conditions.

In the last years, other tissue parameters have started to be assessed by optical means, to investigate breast both in physiologic conditions^15^,^16^ and in the presence of cancer, In particular, it was shown that malignant breast tissue is characterized by higher water and lower lipid content than healthy tissue^17^,^18^,^19^, in agreement with the higher water-to-fat ratio that is measured by magnetic resonance spectroscopy and imaging in tumors^20^,^21^. Moreover, scattering parameters have successfully been assessed to investigate the microscopic structure of tissue^22^,^23^,^24^.

Several *in vivo* studies performed in the past to characterize breast lesions have compared optically derived properties of breast cancer to healthy breast tissue, but not to benign breast lesions^25^. Thus, information obtained on the sensitivity may not be accurate and the specificity of the proposed techniques could not be estimated. More recently, some groups have started to work on the optical discrimination between malignant and benign breast lesions either looking directly at the optical properties (absorption, scattering, and refractive index)^26^,^27^ or combining blood parameters with scattering related parameters^28^,^29^. Very recently, MRI-guided diffuse optical tomography has also been proposed, exploiting MRI imaging as morphological prior to provide more accurate and extensive tissue characterization, including blood, water, lipids and scattering parameters^30^. Our research for the non-invasive characterization of biological tissues has always been based on the time domain approach^31^,^32^,^33^. Short (picosecond) light pulses are injected into the tissue and the re-emitted pulses are detected after propagation through the medium. The optical properties (absorption and scattering) of tissue strongly affect both the amplitude and the shape of the light pulses. Available models of light propagation (*e.g.*, the diffusion theory) account for such dependence, and can thus be exploited to derive the optical properties of tissue from the features of the detected pulses. Over the years, we have progressively extended the spectral range of observation of our laboratory set ups and clinical instruments to achieve a more thorough characterization of tissue, and in particular of breast tissue, quantifying *in vivo* not only blood parameters, water and lipids, but also collagen^34^,^35^,^36^.

The structural protein collagen is involved in tumor development and progression^37^, and could prove to be a useful biomarker of malignant breast lesions. Desmoplasia, the increased deposition and cross linking of the collagen matrix, occurs as a response of the surrounding stroma to cancer development^38^. Moreover, recently it has been observed that tumor invasiveness can affect collagen density and alignment^39^,^40^ and that increased stromal collagen deposition correlates with metastases^41^. Specifically, increased collagen density markedly affects matrix stiffness, which determines the number and nature of binding sites available for neoplastic cell attachment and migration, thus affecting the spreading of metastatic cells^42^. Keeping these observations into account, we decided to include collagen content in the set of parameters exploited to achieve discrimination between malignant and benign breast lesions. Recently, we have reported preliminary data on the average differences between breast lesions and healthy breast tissue for what concerns both absorption properties and tissue composition^19^. As a further step, the present study is an initial investigation on the potential of optically derived tissue composition for the non-invasive discrimination between malignant and benign breast lesions, and on the possible future use of time domain diffuse optical imaging as an adjunct to existing modalities to reduce the overall false positive rate in the detection of breast cancer.

## Results and Discussion

### Imaging of absorption properties and tissue composition

**Figure 1:**
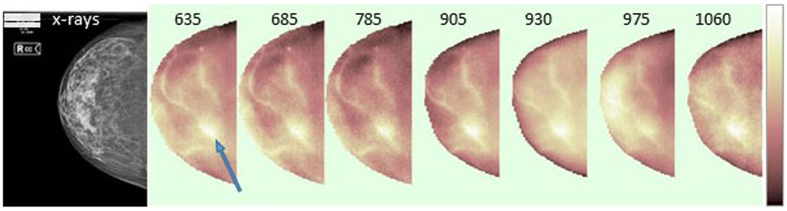
Example of absorption maps at the 7 wavelengths. Right cranio-caudal view of the patient #117 with a fibroadenoma (10 mm size) in the lower-outer quadrant. From left to right, x-ray image and Δ*μ*_*a*_ maps at the 7 wavelengths (635–1060 nm). A blue arrow points to the lesion location. The color-bar range (cm^−1^) for Δ*μ*_*a*_ maps is: −0.39 to 0.052 (635 nm), −0.24 to 0.056 (685 nm), −0.20 to 0.059 (785 nm), −0.33 to 0.05 (905 nm), −0.53 to 0.11 (930 nm), −0.32 to 0.046 (975 nm), −0.34 to 0.089 (1060 nm).

**Figure 2:**
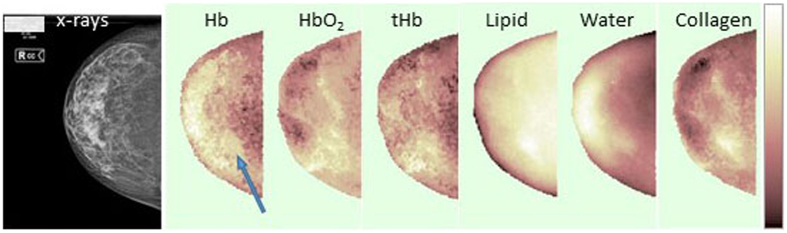
Example of tissue composition maps. Right cranio-caudal view of the patient #117 with a fibroadenoma (10 mm size) in the lower-outer quadrant. From left to right, x-ray image and Δ*C*_*i*_ maps of the main breast constituents (*Hb, HbO*_*2*_, *tHb*, lipid, water and collagen). A blue arrow points to the lesion location. The color-bar range for Δ*C*_*i*_ maps is: −15 to 30 (Δ*Hb* (μM)), −15 to 40 (Δ*HbO*_*2*_ (μM)), −10 to 65 (Δ*tHb* (μM)), −99.5 to 31.5 (ΔLipid (mg/cm^3^)), −200.0 to 325 (ΔWater (mg/cm^3^)), −70 to 140 (ΔCollagen (mg/cm^3^)).

The perturbative approach described in the subsection “Data analysis” allows us to build maps displaying the spatial variation of tissue absorption properties at each of the 7 wavelengths as well as the spatial variation of the concentration of each of the 5 tissue constituents. Figures 1 and 2 show a visual example of the results obtained applying the perturbative approach to optical data collected at 7 wavelengths from a breast with a fibroadenoma (10 mm-diameter). Specifically, Fig. 1 displays the spatial distribution of the absorption properties at the 7 wavelengths by mapping the absorption difference *∆μ*_*a*_ between each image location and the average properties in the same breast. The absorption images can provide qualitative pieces of information on breast tissue. Superficial blood vessels are clearly localized, especially at shorter wavelengths (635–785 nm), where hemoglobin is characterized by stronger absorption. The retroareaolar region, corresponding to denser tissue in the x-ray image, is characterized by intense absorption at 975 nm (wavelength of maximum water absorption), to indicate high water content, as expected in fibro-glandular tissue, while the retromammary fat has lower absorption, consistent with the lower water content of adipose tissue. Instead, 930 nm matches the absorption peak of lipids. The corresponding image appears more uniform and brighter than images collected at other wavelengths, revealing a fatty breast, again in agreement with the x-ray image, which classifies the breast density as BI-RADS (Breast Imaging Reporting and Data System) category 2. Finally, the lesion location appears markedly absorbing at all wavelengths, and especially at short ones. Beside suggesting a high blood content, it is difficult to make any consideration on lesion composition based on *∆μ*_*a*_ maps.

Conversely, information on tissue composition and physiologic blood parameters is available from Fig. 2, which displays concentration differences *∆C*_*i*_ (for *Hb, HbO*_*2*_, *tHb*, water, lipid and collagen), between each image location and the average breast properties. The hemoglobin maps show the higher vascularization and lower oxygenation level of the mammary gland as compared with retromammary fat tissue. The water map confirms high content in the mammary gland and lower content in inner adipose tissue. As expected, lipids show the opposite trend. Collagen is present at high concentration at the lesion location and in the surrounding area. The lesion location is also characterized by higher water content than the surrounding tissue, even though peak values are reached in the mammary gland, and by hemoglobin content higher than average, but again not maximum.

### Analysis of tissue composition and its relationship to breast lesion types

Observations similar to the ones reported in the previous subsection are often common when composition maps are analyzed to characterize breast lesions, both malignant and benign ones, with respect to healthy tissue. Moreover, in a previous preliminary study on a smaller number of subjects we observed average difference in collagen content and *HbO*_*2*_ between malignant and benign lesions^19^. Specifically, both lesion categories had higher collagen and *HbO*_*2*_ than healthy tissue, but on average the difference was bigger for malignant lesions than for benign ones.

**Figure 3:**
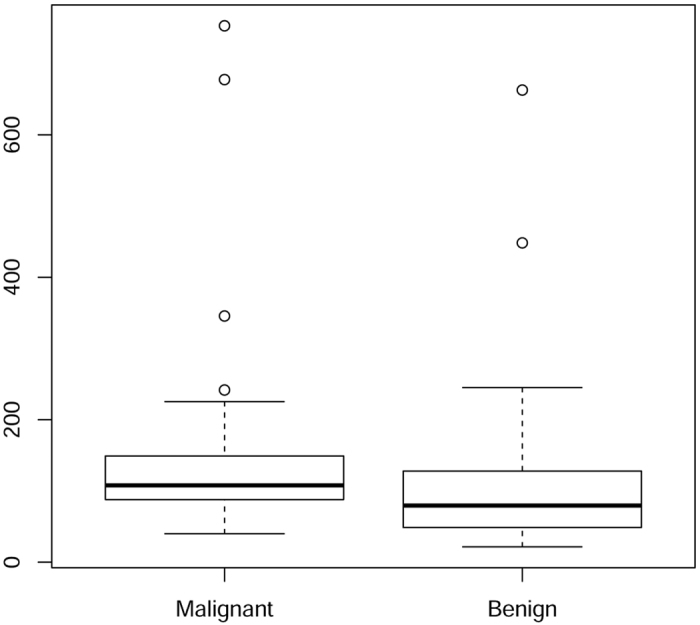
Distribution of tissue composition in malignant and benign lesions. Flanked box plot of the Euclidean distances in R^5^ between the vectors of tissue composition and the related means in the two populations of patients affected by malignant and benign lesions, respectively.

To further investigate the situation, in the present study we considered the multivariate joint distribution of tissue composition (*Hb, HbO*_*2*_, lipid, water, and collagen) in breast lesions with respect to the healthy tissue in the same breast. A two sample non parametric permutation test on the multivariate comparison of the means did not detect a significant difference between the mean concentration vectors of malignant and benign samples (*p*-value = 0.58). If individual tissue constituents are considered, only mean collagen concentrations show significant differences (Mann-Whitney test, *p*-value = 0.033). On the other hand, a permutation test that compares the two variance-covariance matrices showed a highly significant difference (*p*-value = 0.017). Figure 3 displays the flanked box plot of the Euclidean distances in R^5^ between the vectors of tissue composition and the related means in the two populations of patients affected by malignant and benign lesions, respectively. A Mann-Whitney non-parametric comparison test assessed a significant difference between the two distributions (*p*-value = 0.016).

Information on several risk factors for breast cancer is generally available as part of the patient’s history. Thus, we decided to include it when performing classification analysis for lesion discrimination, which was thus based on tissue composition (*Hb, HbO*_*2*_, lipid, water, and collagen) and patient’s anamnesis (age, BMI, familiarity for breast cancer, number of children, use of oral contraceptives and use of preventive Tamoxifen). To account for the inherently stochastic nature of the method, the Discrete AdaBoost procedure was applied 20 times, yielding the following average performances: misclassification rate = 16.5 ± 1.6%, sensitivity = 87.7 ± 1.98%, and specificity = 78.7 ± 3.0%. The AUC (Area Under the Curve) was 0.917. Interestingly, the proposed method correctly classified 3 malignant lesions (invasive ductal carcinomas, confirmed by histology) that were not detected on x-ray mammography (even though their size varied between 18 and 45 mm) and were classified as suspicious on US (BI-RADS 4). This seems to suggest that tissue composition may provide information useful for lesion classification, and not available when only morphology is assessed.

**Figure 4:**
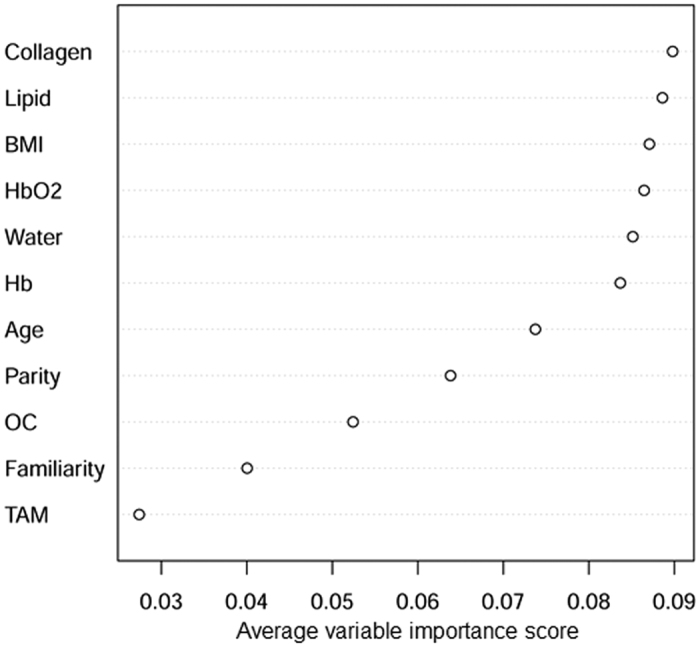
Weight of parameters from tissue composition and patient’s anamnesis in lesion discrimination. Average variable importance rank for lesion discrimination with the Discrete AdaBoost procedure exploiting tissue composition and anamnesis information.

The more frequently a variable is selected for boosting, the more likely it contains useful information for classification. The base classifiers employed are classification/regression trees, so the importance of each variable is computed using a measure of node impurity decrease. Figure 4 reports the average variable importance rank. Apart from BMI that ranks third, tissue constituents generally show higher informative content than other covariates. Specifically, collagen turned out to be the most valuable variable for lesion discrimination, supporting our initial guess on its potential role. Lipid content also proved to be a decisive classifier. In heathy breast tissue collagen and lipid contents show a marked negative correlation (Pearson correlation coefficient −0.78), to indicate a negative correlation between the adipose tissue fraction and the stromal, fibroglandular fraction^43^. When malignant lesions are compared to healthy tissue, the difference in collagen content correlates negatively with the difference in lipid content (Pearson coefficient −0.271, *p*-value 0.026), while no significant correlation is observed for benign lesions (Pearson coefficient −0.079, *p*-value 0.572). The different relationship between collagen and lipids in the different lesion types is in agreement with the fact that they provide independent information for lesion discrimination, as shown by the top positioning of both of them in the variable importance rank.

### Analysis of absorption properties and their relationship to breast lesion types

**Figure 5:**
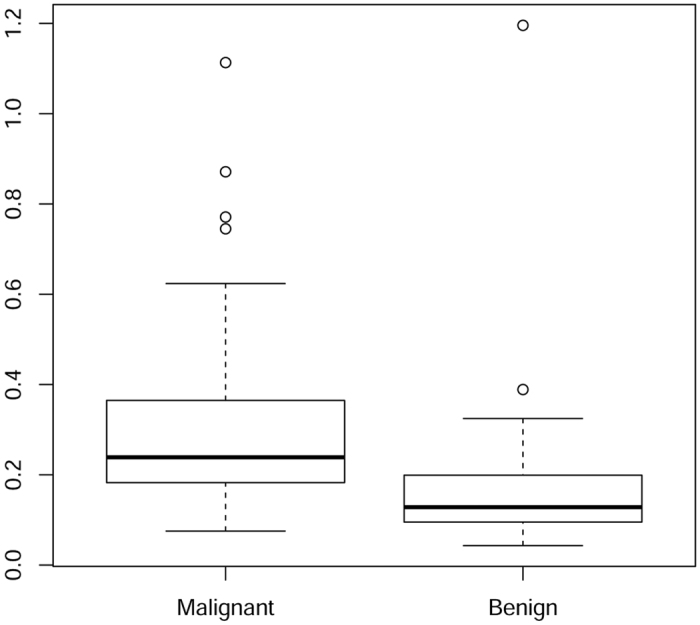
Distribution of tissue absorption in malignant and benign lesions. Flanked box plot of the Euclidean distances in R^7^ between the vectors of absorption differences and the related means in the two populations of patients affected by malignant and benign lesions, respectively.

The absorption properties of tissue are evaluated at 7 wavelengths (635 nm, 685 nm, 785 nm, 905 nm, 930 nm, 975 nm, 1060 nm) as a preliminary step for the estimate of tissue composition. The Beer law, as expressed in equation (2), is then exploited to estimate the concentration of tissue constituents. This second step of data analysis introduces further assumptions – *e.g.*, the knowledge of the spectra of tissue constituents contributing to the overall absorption. As a result, it might increase the error affecting the relationship of optically derived parameters with the pathophysiology tissue aspects, and reduce their effectiveness in discriminating breast lesions. Thus, we have analyzed also the multivariate joint distribution of absorption differences between lesion (malignant or benign) and healthy tissue. The results are consistent with what already reported in the case of tissue composition. Based on a two sample non parametric permutation test on the multivariate comparison of the means, the mean vectors of absorption differences estimated on malignant samples are not significantly different from those obtained on benign samples (*p*-value = 0.53). Conversely, a permutation test that compares the two variance-covariance matrices shows a highly significant difference (*p*-value = 0.010). Moreover, the distributions of the Euclidean distances in R^7^ between the vectors of absorptions and the related means are significantly different in the two populations of patients affected by malignant and benign lesions, as appears from the flanked box plot reported in Fig. 5 and confirmed by a Mann-Whitney non-parametric comparison test (*p*-value < 0.0001).

**Figure 6:**
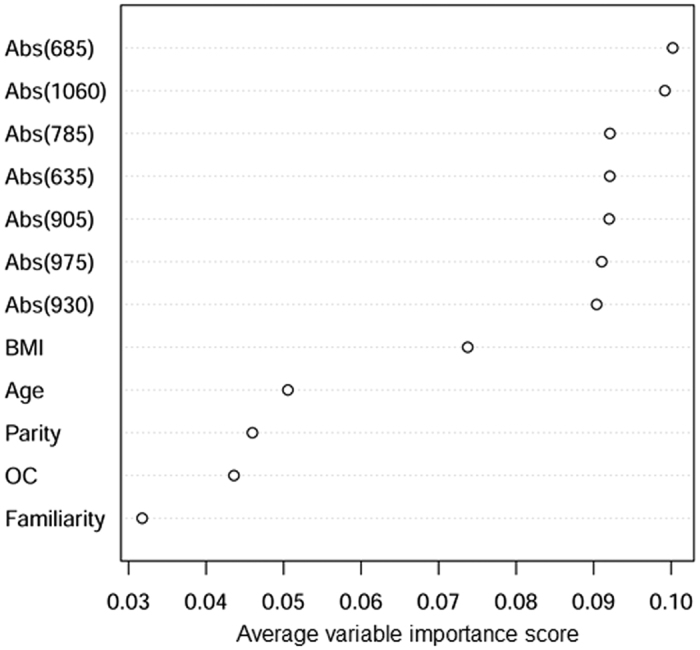
Weight of absorption properties and parameters from patient’s anamnesis in lesion discrimination. Average variable importance rank for lesion discrimination with the Discrete AdaBoost procedure exploiting tissue absorption properties and anamnesis information.

**Figure 7:**
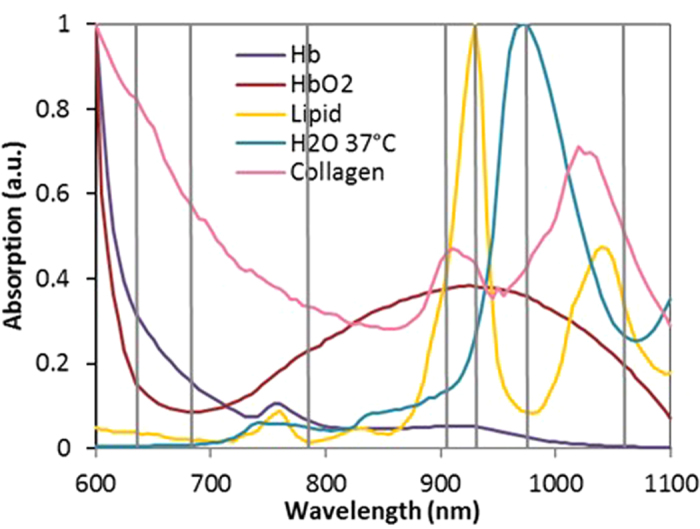
Absorption spectra of the major tissue constituents in the red-near infrared range. The vertical lines identify the 7 wavelengths where measurements are performed.

The classification algorithm was also applied to the absorption differences at 7 wavelengths to test whether improved results could be obtained operating directly on absorption data. The variable importance ranking reported in Fig. 6 shows that the absorption properties at any of the 7 wavelengths are more important than risk factors known from the patient’s anamnesis, and that measurements performed at 685 and 1060 nm are the most effective for lesion discrimination. Based on the absorption line shape of the major tissue absorbers, displayed in Fig. 7, it can be understood that 685 and 1060 nm are both mostly sensitive to collagen content, in agreement with the fact that collagen content is also the most important element for lesion discrimination when tissue composition is considered, as discussed above. Similarly, the performance of the Discrete Boosting algorithm was not significantly different when absorption differences were considered instead of concentration differences, yielding (mean ± SD over 20 repeated runs) misclassification rate = 17.8 ± 2.7%, sensitivity = 80.5 ± 2.3%, and specificity = 84.1 ± 5.5%. The AUC (Area Under the Curve) was 0.921. Hence, at this initial stage of the investigation, we preferred to deal with constituent concentrations, which allow a more straightforward interpretation and link to pathophysiology.

### Limitations of the study

This study was performed to obtain preliminary knowledge on the potential effectiveness of optically derived tissue composition for two-class (malignant/benign) classification of breast lesions. Some limitations of the study should be considered in evaluating the results. The analysis of the present limitations can also provide useful hints for the next steps of the work.

First, due to the limited number of cases that were available for the retrospective analysis (45 malignant and 39 benign), we have included in the “benign” category different types of lesions, which we can reasonably expect to have significantly different composition (*e.g.*, cysts and fibroadenomas). Similar consideration holds for lesions categorized as “malignant”. Working on more homogeneous categories might allow one to better identify the best classifiers, eventually leading to an optimized classification.

Second, limited information was available on several parameters (*e.g.*, duration and end date of use of oral contraceptives (*OC*) and preventive Tamoxifen (*TAM*)). Moreover, only 3 subjects took preventive Tamoxifen. Actually, this may certainly have contributed to make the use of preventive Tamoxifen the least important variable for lesion classification.

Third, collagen proved to be of high relevance for the discrimination of malignant lesions. We estimated the amount of collagen in breast lesions from the non-invasive measurement of tissue absorption and from the knowledge of the absorption properties of each tissue constituent. We had previously assessed the absorption spectrum of collagen type I^34^, which was exploited in the present study. Several types of collagen are present in breast tissue Moreover, it has been reported that new collagen species form during tumor progression. More generally, stromal composition and organization change significantly, and such changes seem distinctive of carcinoma development, not being observed in non-tumoral desmoplastic reactions (*e.g.*, in mammary dysplasia)^44^. Thus, the discrimination performance of our model might be improved including collagen types other than type I in the data analysis. In particular, collagen type V might prove highly relevant, as its amount in ductal invasive carcinoma reaches 10%, while less than 1% is present in normal breast tissue^45^,^46^. Further, collagen is the most abundant protein in breast, but other constituents – not yet optically characterized so far – could be considered (*e.g.*, elastin). More in general, further refinement of the optical spectra of all basic tissue absorbers might help tissue classification.

A final limitation resides in the hypothesis underpinning the perturbation model for data analysis. As extensively discussed elsewhere^19^,^47^, these are mainly the assumptions of a localized small lesion of a given volume and position immersed in an otherwise homogeneous medium, which is a simplifying approximation of the actual scenario. Truly 3D tomographic approaches, even better if exploiting *a priori* knowledge on lesion location obtained from another co-registered imaging modality (*e.g.*, US, x-ray mammography), could help in this respect.

## Summary and Conclusions

Time domain diffuse optical spectroscopy provides quantitative information on tissue absorption and, in turn, on tissue composition. Specifically, measurements performed at 7 wavelengths (635–1060 nm) provided differences in absorption properties at the 7 wavelengths where measurements were performed and in tissue composition (oxyhemoglobin (*HbO*_*2*_), deoxyhemoglobin (*Hb*), water, lipid, and collagen) between lesion and average healthy tissue in the same breast for 45 malignant and 39 benign lesions. We have applied classification analysis (namely, the Discrete AdaBoost procedure) to discriminate non-invasively malignant from benign breast lesions based on optically derived tissue composition or absorption properties and information obtained from the patient’s anamnesis, achieving average sensitivity in the range 81–88% and specificity 79–84%.

In this initial test, collagen content, closely followed by lipid content, proved the most important parameter for lesion classification, based on tissue composition. In agreement, when the spectrally resolved absorption properties of tissue are considered, 635 and 1060 nm, which are mostly sensitive to collagen, appear most effective for discrimination.

This study represents an initial step in the investigation on how diffuse optics could contribute to the non-invasive *in vivo* classification of breast lesions. More extensive studies are now needed and the limitations of the present study, discussed in the text, will have to be removed or minimized to allow a clear identification of the best way to apply the proposed method, and a reliable evaluation of its potential.

## Methods

### Instrument set-up

Our optical mammograph operates at 7 red-near infrared wavelengths, in the time domain, in transmittance geometry, *i.e.*, in the same geometry as x-ray mammography, but with milder breast compression (Supplementary Figure S1). The instrument is portable (50 cm W × 80 cm D × 140 cm H), mounted on wheels, and approved for use in a clinical environment.

The schematics of the set-up is shown in Supplementary Figure S2. Light sources are 7 pulsed diode lasers (LDH-P-XXX, PicoQuant, Germany, where XXX denotes the nominal wavelength in nanometers) emitting at 635, 685, 785, 905, 930, 975 and 1060 nm, with average output power of ~1–5 mW, temporal width of ~150–400 ps (full width at half maximum, FWHM), and repetition rate of 20 MHz. All laser heads are controlled by a single driver (PDL-808 “Sepia”, PicoQuant, Germany). The emitted pulses are properly delayed by means of graded index optical fibers, and combined into a single coupler. Circular variable neutral density filters allow the optimization of the illumination power independently at each wavelength. The breast is mildly compressed between parallel antireflection-coated tempered glass plates, causing no significant discomfort to the patient. A fiber bundle collects the pulses transmitted along the line of sight. Its distal end is bifurcated and guides photons respectively to a photomultiplier tube (PMT) for the detection of wavelengths <850 nm (R5900U-01-L16, Hamamatsu, Japan) and to a PMT for longer wavelengths (H7422P-60, Hamamatsu, Japan). Circular variable neutral density filters in the detection paths control the detected power. Two personal computer boards for time-correlated single photon counting (SPC134, Becker&Hickl, Germany) perform the acquisition of time-resolved transmittance curves at wavelengths shorter and longer than 850 nm, respectively. Depending on wavelength, the instrument response function ranges between 460 and 930 ps (full width at half maximum).

The illumination fiber and collection bundle are scanned in tandem and a feedback on the total number of counts per point restricts the scan to the breast area. Data are stored every millimeter of path, *i.e.* every 25 ms. A complete scan typically requires 5 min.

The compression unit can be rotated by an angle up to 90° in both clock-wise and counter-clock-wise direction, to acquire images in the cranio-caudal (CC), medio-lateral or oblique (OB, at 45°) views.

Further details on the instrument set-up and performances, and on the procedures for data acquisition and analysis are reported in ref. 35.

### Subjects and measurement procedure

Data were collected between 2009 and 2013 from 200 subjects as part of a study on the optical characterization of malignant and benign breast lesions and the non-invasive assessment of breast density by optical means.

Written informed consent was obtained from all subjects, and all protocols were approved by the Institutional Review Board of the European Institute of Oncology. The study was performed in accordance with the Declaration of Helsinki. More generally, all experiments were carried out in accordance with relevant guidelines and regulations.

Only lesions with maximum diameter of at least 10 mm were considered for the optical characterization, resulting in a subset of 45 malignant lesions and 39 benign lesions identified for further analysis. Most frequent malignant lesions included in the study were ductal invasive carcinomas and lobular invasive carcinomas (33 and 5, respectively), while benign lesions were fibroadenomas and cysts (14 and 6, respectively). Details on lesion type are reported in Supplementary Table S1.

From each subject, four sets of 7-wavelength transmittance images were acquired (*i.e.* CC and OB views of both breasts). For the entire duration of the acquisition, the subject was sitting comfortably.

### Data analysis

#### Estimate of tissue absorption and composition

Light propagation in breast tissue was modeled using the diffusion approximation to the radiative transport theory with extrapolated boundary condition, for a homogeneous infinite slab, which allows the estimate of the average absorption and reduced scattering coefficients from the measurement of time-resolved transmittance data^48^,^49^.

A perturbative approach was applied to optically characterize breast lesions, as described in detail in ref. 19. Briefly, we assume that lesions can be treated as localized absorption perturbations embedded in an otherwise homogeneous diffusive medium (*i.e.*, the healthy tissue in the same breast). The unperturbed (healthy tissue) and perturbed (lesion) time-resolved transmittance curves, *T*_0_(*t*) and *T(t*), respectively, are linked through the modified Lambert-Beer’s law:


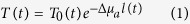


where *l(t*) is the mean pathlength traveled in the perturbation by photons detected at time *t*, while *∆μ*_*a*_ represents the absorption difference between perturbation (lesion) and unperturbed background (average breast tissue). Time gating (*i.e.* temporal binning) of the experimental time-resolved curves was exploited to improve the signal-to-noise ratio. Specifically, data were analyzed dividing the transmittance curves in 10 equal-counts time windows^50^. The 8^*th*^ time window was chosen to provide information on tissue absorption, as it collects late photons, which are sensitive to absorption and less affected by possible scattering variations. The unperturbed curve *T*_0_(*t*) was obtained as an average over an area of the breast image that excludes boundaries and marked inhomogeneities, but still includes most of the breast^19^. The perturbed curve *T(t*) was obtained from an area centered on the corresponding perturbation (lesion) position. A “lesion area” of 9 × 9 mm^2^ was selected for lesion diameters >15 mm, otherwise it was set to 5 × 5 mm^2^. The pathlength *l(t*) was derived from the average breast optical properties, applying an 8^th^ order perturbation approach as described in refs 47, 51, and exploited to estimate *∆μ*_*a*_ from the measurement of *T*_0_(*t*) and *T(t*).

Once *∆μ*_*a*_ had been appraised and knowing the extinction coefficient of the main constituents of breast tissue, the Beer law was used to relate the absorption properties at wavelength *λ* to the concentrations of the main tissue constituents:


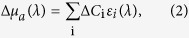


where *ε*_*i*_(*λ*) is the extinction coefficient of the *i*-th constituent at wavelength *λ*, while *∆μ*_*a*_ and *∆C*_*i*_ are the absorption difference and concentration difference, respectively, between lesion and average breast tissue. This procedure allowed us to estimate the concentration variation between lesion and average breast tissue for oxy- and deoxy-hemoglobin (*HbO*_*2*_ and *Hb*, respectively), water, lipid, and collagen.

The perturbation method adopted in the present study relies on the *a priori* knowledge of lesion volume and location. We have always described the lesion as a spherical inhomogeneity located halfway between source and detector position. To approximate its volume, we considered an equivalent sphere based on the maximum lesion diameter obtained by histopathology, when available (*i.e.,* for all malignant lesions and part of the benign lesions), and by x-ray mammography or US elsewhere.

The perturbative approach was applied also to estimate *∆μ*_*a*_ and *∆C*_*i*_ between position (*x, y*) (considered as a potential lesion location) and the average breast tissue, and scanning (*x, y*) over the whole breast extension. This procedure allows us to visualize the spatial variation of both absorption properties and tissue composition. Specifically, images were generated for *Hb HbO*_*2*_, blood volume (total hemoglobin content *tHb* = *Hb* + *HbO*_*2*_), water, lipid and collagen content as well as for the absorption properties at the 7 wavelengths.

#### Statistical analysis

We studied the multivariate joint distribution of optically derived tissue composition of malignant and benign lesions, in terms of *Hb* and *HbO*_*2*_, lipid, water, and collagen. In particular, a two-sample non parametric permutation test on the multivariate comparison of the means was applied to detect differences between the mean concentration vectors for malignant and benign lesions^52^. A permutation test (Mantel test) was used to compare the two variance-covariance matrices^53^. The Euclidean (straight-line) distances were calculated in R^5^ between the vectors of tissue composition and the related means in the two populations of patients affected by malignant and benign lesions, respectively. The interquartile ranges of the features considered in the calculation of the Euclidean distances were all of the same order of magnitude. A Mann-Whitney non-parametric comparison test was made to assess whether a significant difference exists between these two distributions.

The same tests were also performed to compare the distributions of absorption differences *∆μ*_*a*_ measured at each of the 7 wavelengths in case of malignant lesions with the corresponding distributions for benign lesions.

To achieve lesion discrimination, we relied on both optically derived tissue composition (*Hb* and *HbO*_*2*_, lipid, water, and collagen) or absorption properties and on information known from the patient’s demographics and anamnesis: age, body mass index (*BMI*), parity (number of children, ranging from 1 to 4), family history of breast cancer (first-degree), use of oral contraceptives (*OC*), and use of preventive Tamoxifen (*TAM*). In order to discriminate malignant from benign breast lesions, classification analysis was performed. Specifically, we implemented the most common version of the AdaBoost procedure with decision trees, the so called Discrete AdaBoost^54^. Boosting is a machine learning algorithm and works by sequentially applying a classification algorithm to reweighted versions of the training data and then taking a weighted majority vote for the classifiers thus produced. Assuming that *f*_*m*_(*x*) are classifiers producing values of plus or minus 1, we define:


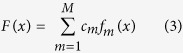


where *c*_*m*_ are suitable constants. The corresponding prediction is given by *sign(F(x*)). The Adaboost procedure trains the classifiers *f*_*m*_(*x*) on weighted versions of the training sample, giving higher weight to cases that are currently misclassified. The base classifiers are classification/regression trees, so the importance of each variable is computed using a measure of node impurity decrease. This step is repeated for a sequence of weighted samples, and then the final classifier is defined to be a combination of the classifiers from each stage. The number of iterations was set to 50, the default in the ada package. This value guarantees the algorithm convergence. The algorithm of the Discrete AdaBoost is summarized in Table 1, where *E*_*w*_ represents the expectation over the data with weights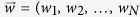). At each iteration, the algorithm increases the weights of the observations misclassified by *f*_*m*_(*x*) by a factor that depends on the weighted error. To avoid building a classifier that would be extremely adherent to the reference sample, this method relies on applying a random sampling strategy (bagging): at each iteration a sample is randomly drawn from the dataset, and used to train weak classifiers. Thus, the final results are subject to stochasticity. To account for that, we repeated the procedure 20 times, and evaluated its performance in terms of mean ± SD misclassification rate, sensitivity, and specificity.

Specifically, we fitted the Discrete Adaboost using the ada package in R^55^,^56^.

## Additional Information

**How to cite this article**: Taroni, P. *et al*. Non-invasive optical estimate of tissue composition to differentiate malignant from benign breast lesions: A pilot study. *Sci. Rep.* **7**, 40683; doi: 10.1038/srep40683 (2017).

**Publisher's note:** Springer Nature remains neutral with regard to jurisdictional claims in published maps and institutional affiliations.

## Supplementary Material

Supplementary Information

## Figures and Tables

**Table 1:** Algorithm of the Discrete AdaBoost.

Start with weights *w*_*i*_ = 1/*N*
Repeat for *m* = 1, 2, …, *M*:
-Fit the classifier *f*_*m*_(*x*) ∈ {−1, 1} using weights *w*_*i*_ on the training data
-Compute 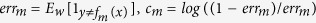
-Set 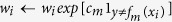 and renormalize so that 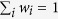
Output the classifier