# Supplementary material for: Non-invasive optical estimate of tissue composition to differentiate malignant from benign breast lesions: A pilot study
Source: Sci Rep. 2017 Jan 16;7:40683. doi: 10.1038/srep40683 (PMC5238417; doi:10.1038/srep40683)
Supplement: Supplementary Information [file srep40683-s1.pdf]

Non-invasive optical estimate of tissue composition to differentiate malignant from benign breast lesions: A pilot study

Paola Taroni, Anna Maria Paganoni, Francesca Ieva, Antonio Pifferi, Giovanna Quarto, Francesca Abbate, Enrico Cassano, and Rinaldo Cubeddu

Supplementary information

Supplementary Table S1: **Lesion types and grouping** as malignant/benign.

Supplementary Figure S1: **Photo of the optical mammograph**. The collection of an image takes on average about 5 min. Thus, during data acquisition the subject is sitting comfortably.

Supplementary Figure S2: **Schematics of the optical mammograph**. NIR, near-infrared; PMT, photomultiplier tube; TCSPC, time-correlated single photon counting. Reproduced from Ref. [35].

Supplementary Table S1: Lesion types and grouping as malignant/benign.

Patient ID	Breast	Lesion group	Lesion type
13	L	Benign	Phyllodes tumor
15	R	Benign	Cyst
19	R	Malignant	Invasive lobular carcinoma
22	R	Benign	Papilloma
30	L	Malignant	Invasive ductal carcinoma
40	R	Benign	Fibroadenoma
52	R	Benign	Adipose lobule
53	R	Benign	Fibroadipose nodule
55	L	Benign	Fibrosis
60	L	Malignant	Invasive ductal carcinoma
61	R	Malignant	Invasive ductal carcinoma
70	L	Benign	Fibroadenoma/Fibroadenolipoma
74	L	Malignant	Invasive ductal carcinoma
80	L	Benign	Cyst
86	R	Malignant	Invasive ductal carcinoma
88	L	Malignant	Invasive mucinous carcinoma
91	L	Malignant	Invasive lobular carcinoma
93	L	Malignant	Ductal intraepithelial neoplasia, grade II
96	R	Malignant	Invasive ductal carcinoma
97	R	Malignant	Invasive ductal carcinoma
99	L	Malignant	Invasive ductal carcinoma
100	L	Malignant	Carcinoma
101	L	Malignant	Invasive ductal carcinoma
102	L	Malignant	Invasive ductal carcinoma
103	L	Malignant	Invasive lobular carcinoma
105	L	Malignant	Invasive ductal carcinoma
107	L	Benign	Fibrosis
108	L	Malignant	Invasive ductal carcinoma
110	R	Malignant	Invasive ductal carcinoma
111	R	Benign	Benign nodule
112	R	Malignant	Invasive lobular carcinoma
113	R	Malignant	Invasive mucinous carcinoma
116	L	Benign	Proliferative fibrocystic disease
117	R	Benign	Fibroadenoma
117	L	Malignant	Invasive ductal carcinoma
118	L	Malignant	Ductal intraepithelial neoplasia, grade II
119	R	Malignant	Invasive ductal carcinoma
119	L	Benign	Fibroadenoma
122	L	Malignant	Invasive ductal carcinoma
124	L	Malignant	Invasive ductal carcinoma
126	L	Benign	Papilloma
128	R	Malignant	Invasive ductal carcinoma
130	R	Malignant	Invasive ductal carcinoma
131	R	Benign	Fibroadenoma
140	L	Malignant	Invasive ductal carcinoma
141	R	Benign	Fibroadenoma

Patient ID	Breast	Lesion group	Lesion type
152	L	Malignant	Invasive ductal carcinoma
155	L	Benign	Cyst
157	L	Benign	Cyst
158	R	Malignant	Micropapillary carcinoma
160	R	Malignant	Invasive ductal carcinoma
161	R	Malignant	Invasive ductal carcinoma
162	R	Benign	Fibroadenoma
166	L	Malignant	Invasive ductal carcinoma
167	L	Benign	Fibroadenoma
168	R	Malignant	Invasive ductal carcinoma
169	R	Benign	Phyllodes tumor
173	L	Malignant	Invasive ductal carcinoma
175	R	Malignant	Invasive mucinous carcinoma
176	R	Benign	Fibroadenoma
176	L	Malignant	Invasive lobular carcinoma
177	R	Malignant	Invasive ductal carcinoma
179	L	Malignant	Invasive ductal carcinoma
182	L	Benign	Fibrocystic disease
183	L	Benign	Adipose nodule
187	L	Malignant	Invasive ductal carcinoma
193	L	Malignant	Invasive ductal carcinoma
194	R	Malignant	Invasive ductal carcinoma
196	L	Benign	Cyst
199	R	Malignant	Invasive ductal carcinoma
200	R	Benign	Fibroadenoma
201	R	Benign	Fibroadenoma
204	L	Benign	Fibroadenoma
205	L	Malignant	Invasive ductal carcinoma
208	L	Benign	Fibroadenoma
209	R	Benign	Phyllodes tumor
210	R	Benign	Fibroadenoma
210	L	Benign	Benign nodule
211	R	Benign	Papilloma
212	L	Benign	Fibrocystic disease
214	R	Benign	Lipoma
215	L	Benign	Fibrolipoma
216	L	Benign	Cyst
217	R	Benign	Hamartoma

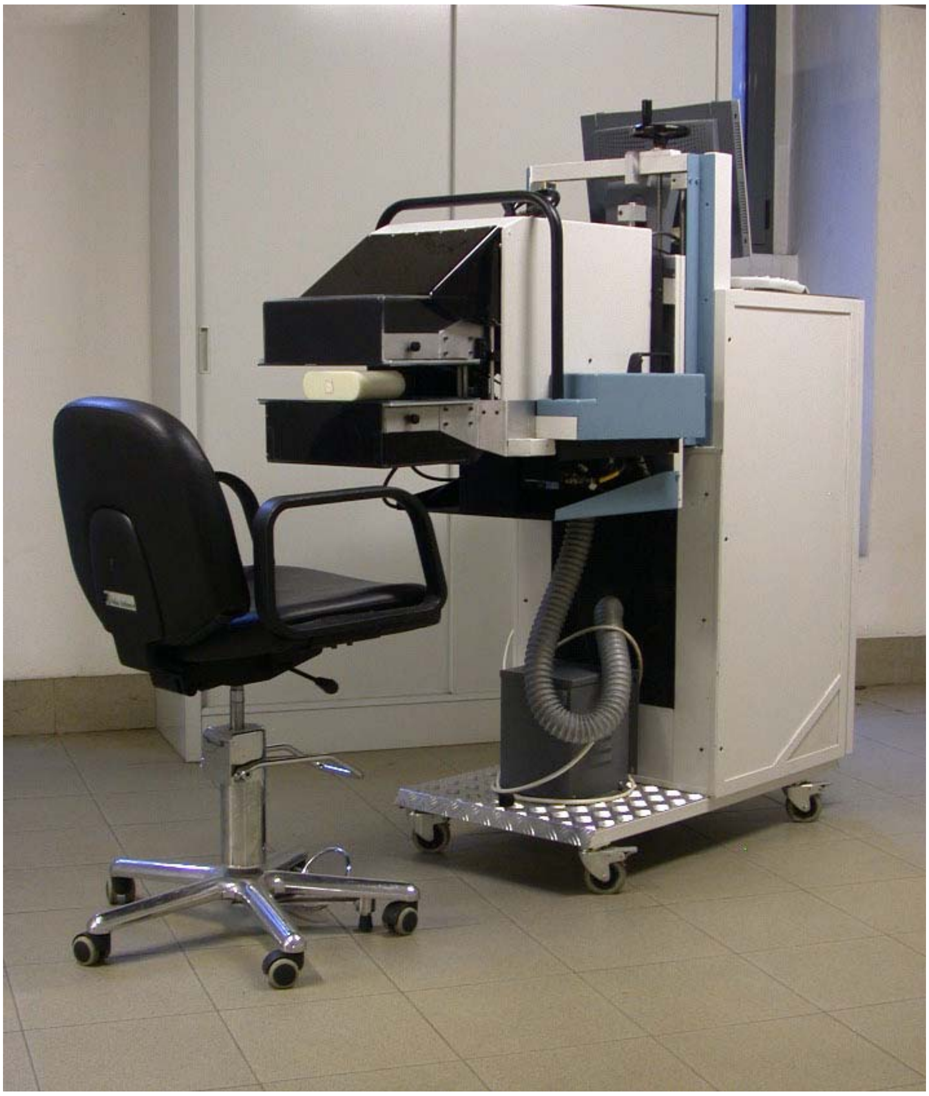

Supplementary Figure S1: **Photo of the optical mammograph**. The collection of an image takes on average about 5 min. Thus, during data acquisition the subject is sitting comfortably.

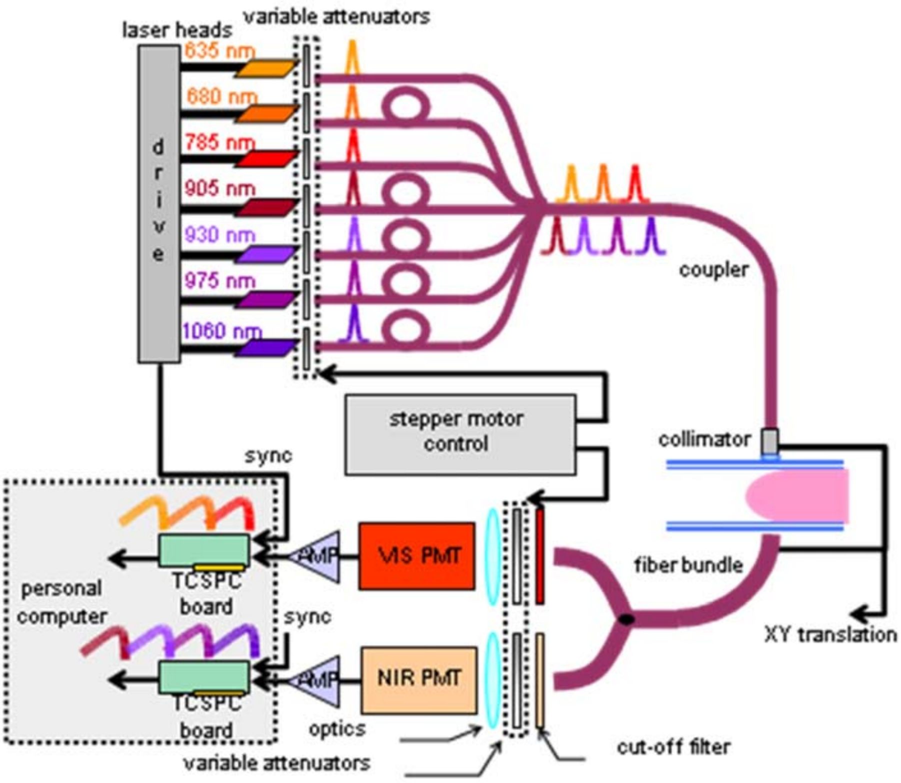

Supplementary Figure S2: **Schematics of the optical mammograph**. NIR, near-infrared; PMT, photomultiplier tube; TCSPC, time-correlated single photon counting. Reproduced from Ref. [34].
